# Reliability and correlates of cross-sectional area of abductor hallucis and the medial belly of the flexor hallucis brevis measured by ultrasound

**DOI:** 10.1186/s13047-018-0259-0

**Published:** 2018-06-07

**Authors:** Penelope J. Latey, Joshua Burns, Elizabeth J. Nightingale, Jillian L. Clarke, Claire E. Hiller

**Affiliations:** 1 Faculty of Health Sciences, The University of Sydney, Sydney, NSW Australia; 2 Paediatric Gait Analysis Service of New South Wales, Sydney Children’s Hospitals Network (Randwick and Westmead), Sydney, NSW Australia

**Keywords:** Ultrasound, Pedobarography, Dynamometry, Intrinsic foot muscles, Arch height, Toe flexor strength

## Abstract

**Background:**

Weakness of the intrinsic foot muscles is thought to produce deformity, disability and pain. Assessing intrinsic foot muscles in isolation is a challenge; however ultrasound might provide a solution. The aims of this study were to assess the reproducibility of assessing the size of abductor halluces (AbH) and the medial belly of flexor hallucis brevis (FHBM) muscles, and identify their relationship with toe strength, foot morphology and balance.

**Methods:**

Twenty one participants aged 26–64 years were measured on two occasions for muscle cross-sectional area using a Siemens Acuson X300 Ultrasound System with 5-13 MHz linear array transducer. Great toe flexor strength was measured by pedobarography, the paper grip test and hand-held dynamometry. Foot morphology was assessed by foot length, truncated foot length, Foot Posture Index (FPI) and dorsal arch height. Balance was measured by the maximal step test. Intra-class correlation coefficients (ICC_3,1_) were used to evaluate intra-rater reliability. Pearson’s correlation coefficients were performed to assess associations between muscle size and strength, morphology and balance measures. To account for the influence of physical body size, partial correlations were also performed controlling for truncated foot length.

**Results:**

Intra-rater reliability was excellent for AbH (ICC_3,1_ = 0.97) and FHBM (ICC_3,1_ = 0.96). Significant associations were found between cross-sectional area of AbH and great toe flexion force measured standing by pedobarography (*r* = .623, *p* = .003),), arch height measured sitting (*r* = .597, *p* = .004) and standing (*r* = .590, *p* = .005), foot length (*r* = .582, *p* = 006), truncated foot length (*r* = .580, *p* = .006), balance (*r* = .443, *p* = .044), weight (*r* = .662, *p* = .001), height (*r* = .559, *p* = .008), and BMI (*r* = .502, *p* = .020). Significant associations were found between cross-sectional area of FHBM and FPI (*r* = .544, *p* = .011), truncated foot length (*r* = .483, *p* = .027) and foot length (*r* = .451, *p* = .040). Significant partial associations were found between AbH and great toe flexion force in standing by pedobarography (*r* = .562, *p* = .012) and FHBM and the FPI (*r* = .631, *p* = .003).

**Conclusions:**

Measuring the cross-sectional area of AbH and FHBM with ultrasound is reproducible. Measures of strength, morphology and balance appear to relate more to the size of AbH than FHBM. After controlling for physical body size, cross-sectional area of AbH remained a significant correlate of great toe flexor strength and might be a useful biomarker to measure early therapeutic response to exercise.

## Background

Intrinsic foot muscle weakness is related to common foot pathologies and deformities [1–4] and may be caused by neuromuscular conditions such as diabetic neuropathy [5, 6] and Charcot-Marie Tooth disease [7, 8]. Reduction in toe flexion strength is associated with an increased risk of falling in older adults [9, 10]. The intrinsic great toe muscle abductor hallucis acts as a dynamic elevator, [11] helps maintain balance in a medio-lateral direction [12] and supports the medial longitudinal arch [13]. Improving toe flexion strength can minimise the effect of foot muscle atrophy induced by disease or deformity, [14, 15] and improve upright dynamic functional movement [16]. The ability to reliably measure the cross-sectional area of the small first ray muscles may be an important early biomarker of treatment strategies for foot muscle weakness.

The toes are stabilised and acted on by both intrinsic and extrinsic foot muscles. Accuracy in evaluating the strength of intrinsic great toe muscles and their specific contribution to dynamic balance, or their relationship to foot morphology remains a challenge [17]. Toe flexion force measures do not distinguish intrinsic from extrinsic foot muscles [18]. Muscle specificity can be determined by size or cross-sectional area; however muscle size does not entirely explain differences in strength [19]. Since the first ray performs as one functional unit, [20] ascertaining if there is an association between the cross-sectional area of abductor hallucis (AbH) and the medial belly of flexor hallucis brevis (FHBM) muscles with measures of toe flexion force may provide a more accurate picture of the role these muscles have in medial longitudinal arch support and great toe muscle weakness.

Imaging cross-sectional area using Computerised Tomography (CT) [21] Magnetic Resonance Imaging (MRI) [22] or ultrasound [23] enables analysis of specific muscles and regions of the foot. Although MRI and CT have a high level of accuracy, [24] they are usually not immediately available in research or clinical practice due to cost. Ultrasound is a non-invasive, non-ionising and inexpensive method of assessing muscle morphology or size. Measuring cross-sectional area using ultrasound of AbH, flexor hallucis brevis, flexor digitorum brevis, quadratus plantae and abductor digiti minimus muscles in supine or prone has been reported as highly reliable [1, 23, 25]. However, previous studies have not scanned the person in an upright position. In a clinical situation with a broad population base there can be limitations on patient’s movement ability. Some patients are unable to turn over from supine to prone or even lie down flat on a treatment table due to various problems such as: severe back problems, [26] obesity, [27] positional vertigo [28] or sarcopenia [29]. Cross-sectional area of the lower limb can also be affected by position [30]. Therefore the scanning position was modified to determine if scanning the medial foot in seated, with the ankle in a mid-range neutral position was as reliable as the supine or prone positions. As scanning the foot on its plantar aspect was impractical with the participant seated, and on reviewing the anatomical pathways of FHB, only the medial fibres of FHB were scanned.

The aims of this study were to assess the reproducibility of assessing the size of abductor halluces (AbH) and the medial belly of flexor hallucis brevis (FHBM) muscles, and identify their relationship with toe strength, foot morphology and balance. Since the cross-sectional area and muscle thickness of the ABH, FHB, flexor digitorum brevis, quadratus plantae and lumbricals have been shown to be associated with toe flexor strength [31] we hypothesised that a decreased size of AbH and FHBM scanned in the seated position would be similarly related to toe flexor weakness. The relationships between muscle size and foot morphology were explored as, despite the understanding that some variability in muscle thickness, [32] size [33] and strength [34] may be attributed to participant characteristics, the effect of foot morphology on muscle size has yet to be determined.

Toe flexion strength has been shown to be important determinant of balance, [35] and is related to increased single leg balance time in older adults [36]. Correspondingly, reduced toe flexion strength has been associated with impaired balance, [37] increased postural sway and reduced functional ability in older adults [38]. More specifically, AbH, flexor digitorum brevis and quadratus plantae muscles increase activity with increasing postural demands and help maintain balance in a medial-lateral direction [12]. Therefore we also hypothesised that a greater cross-sectional area of AbH and FHBM would be associated with better balance.

## Methods

### Participants

Twenty one participants were recruited from the University of Sydney and general population via an advertisement. Participants were healthy adults, 18 to 65 years of age, able to walk barefoot and unaided. Study exclusion criteria were a history of a musculoskeletal or systemic disease (e.g. Diabetes type 2), acute familial or acquired foot problem (e.g. Charcot Marie Tooth Syndrome) or injury affecting foot or lower limb joint motion, foot surgery, or severe foot pain (≥7on a 0–10 point scale).

### Measures and procedures

All participants attended two data collections 2–4 weeks apart. At the first data collection, participant characteristics were recorded, including age, sex, height, weight and dominant foot (determined by asking with which foot the participant kicked a ball). All other measures were taken of the dominant foot three times at each data collection session to determine reliability of testing procedures and the measures used. Data collected at the first session was kept in a locked cabinet until all data collections were completed. The second data collection was completed without the researcher having access to the first data set.

#### Ultrasound

Ultrasound cross-sectional area of AbH and FHBM were measured using a Siemens Acuson X300 Ultrasound System (Siemens Medical Solutions, Inc., Mountain View, California, USA) with 5-13 MHz linear array transducer. Each non-weight bearing ultrasound image was collected with participants seated on a raised plinth with their leg relaxed, knee flexed 90°.

**Fig. 1:**
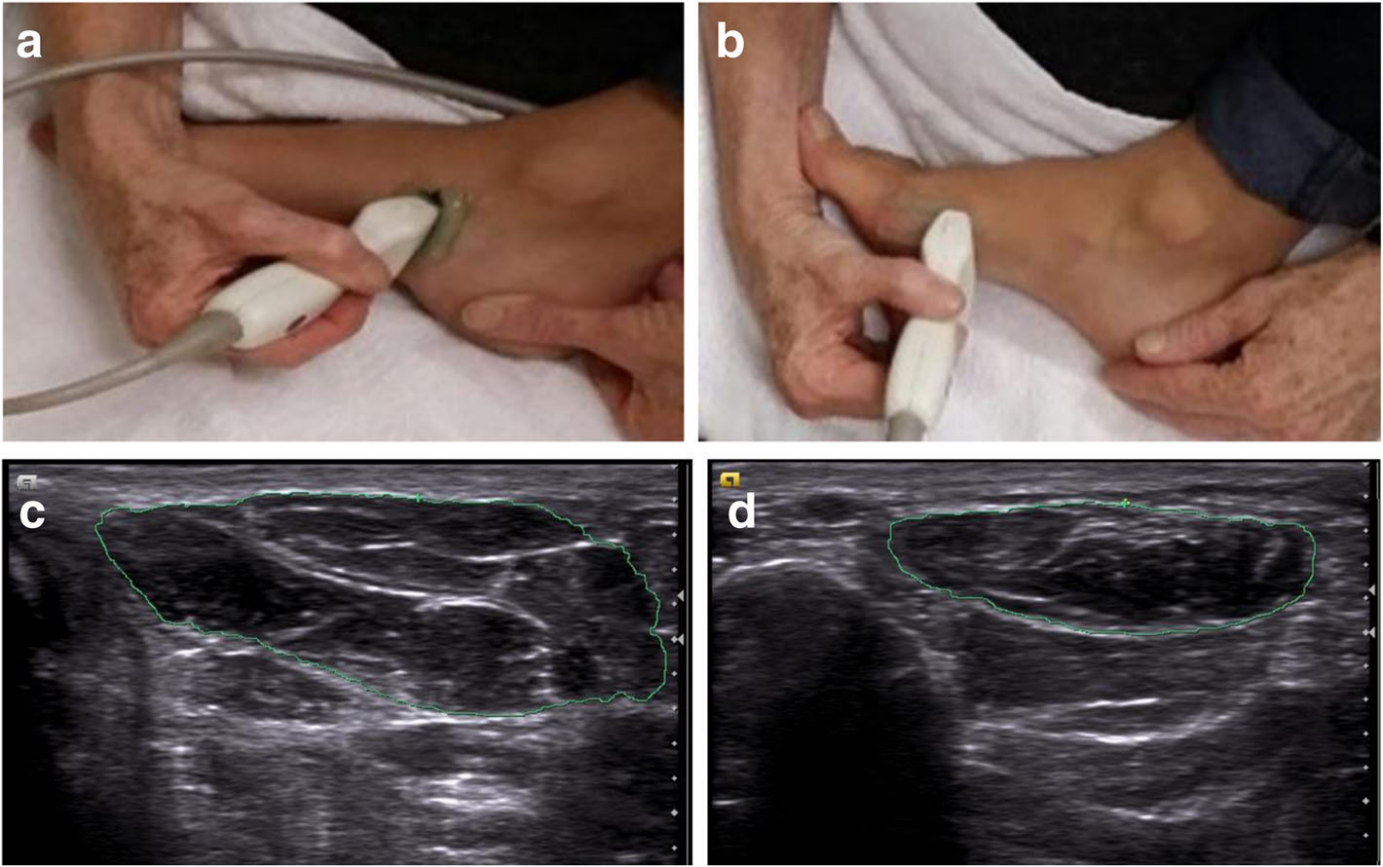
Ultrasound transducer placement, scanned image and outlined circumference. **a** Transducer placement to scan the AbH muscle, **b** Transducer placement to scan the FHBM muscle, **c** Ultrasound image of the cross-sectional area of AbH outlined, **d** Ultrasound image of the cross-sectional area of FHBM outlined

The lateral border of the participant’s stabilised foot rested on the thigh of the seated researcher, with the ankle positioned in neutral. The plantar aspect of the foot faced towards the floor, to allow contiguous transducer access to both the medial and plantar aspects of the foot. To identify the AbH muscle the researcher first palpated, then marked the navicular tubercle. Ultrasound gel was placed between the skin and transducer to remove air artefact and ensure good transducer to skin contact. The transducer was then placed on the navicular tubercle and the long axis of the transducer moved inferiorly in a directly perpendicular line across the mid arch of the medial longitudinal arch to identify AbH in cross section (Fig. 1a, c). To identify the FHBM muscle, the medial sesamoid bone was first palpated, then marked and ultrasound gel placed on the participants’ skin in line with the 1st metatarsal bone. The end of the transducer was used to locate the medial sesamoid bone, and the long axis of the transducer aligned with the longitudinal aspect of the muscle belly. The transducer was moved proximally along the FHBM until only the proximal edge of the medial sesamoid bone and its acoustic shadow could be observed on the image. The thickest part of the muscle was then identified and the transducer was rotated 90° at 50% of transducer length. The transducer was then translated inferiorly towards the plantar aspect of the foot within the coronal plane until a clear image of the FHBM muscle could be visualised. The FHBM was thus scanned perpendicularly to the muscle, to capture its maximal cross-sectional area. This scanning location was on the medial-plantar aspect of the foot, mid metatarsal (Fig. 1b, d). The cross-sectional area was determined by tracing the muscle outline of the scanned images and the area was calculated by the Siemens Acuson program software.

#### Muscle strength

Toe flexor strength of the dominant foot was measured with pedobarography using the Emed® pressure platform, paper grip test and hand held dynamometry. A standing position was used for the Emed® [39] paper grip test and hand held dynamometry measuring devices [4, 18, 40]. The following procedure was repeated for each strength test. The participant was first familiarised with the toe flexor task by passive demonstration of the movement required, followed by active practice until the participant could perform the test correctly. Subsequently, three consecutive contractions of 3 to 5 s for the toe flexor task were recorded. Verbal encouragement was given during each contraction.

**Fig. 2:**
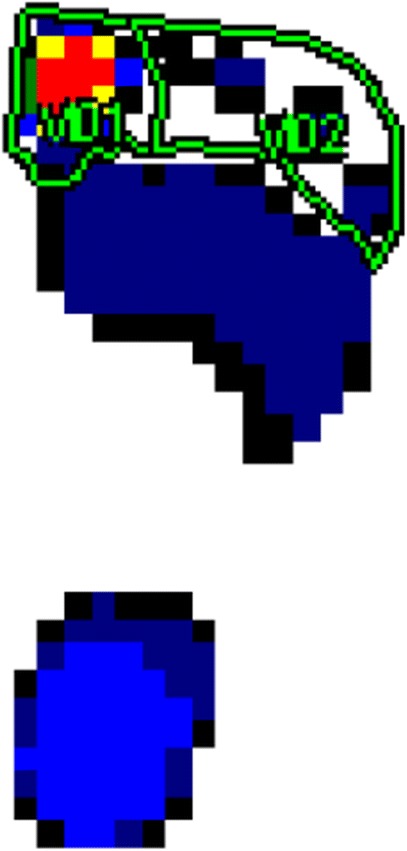
Pedobarography- Emed Pressure Map of standing great toe flexion with 2-toes Mask applied

For the toe flexor testing using the Emed®-AT/2 capacitance pressure distribution platform (Novel GmbH, Munich, Germany), sensor area 360 mm × 190 mm containing 1377 sensors, resolution 2 sensors/cm2 (recording frequency 25 Hz), participants were instructed to press down on the platform as hard as possible using only their great toe. Directions were given to elongate the toes and elevate the mid arch by pressing distal ends of the toes down while keeping their heels on the platform. For both tasks the participant’s torso remained upright with arms crossed in front of their chest, palms up and looking straight ahead. Peak forces were recorded by the software [39]. An Emed® Mask (Novel GmbH, Munich, Germany) was created for the great toes to determine maximal force and mean pressure during the great toe flexor task (Fig. 2).

The procedure for the paper grip test was similar to that for the pressure platform test. Participants stood and were directed to press the great toe, then the lesser toes downwards while attempting to hold a card down with the toes. This was a modified position from de Win’s, and was a pass/fail test of three consecutive attempts [18].

**Fig. 3:**
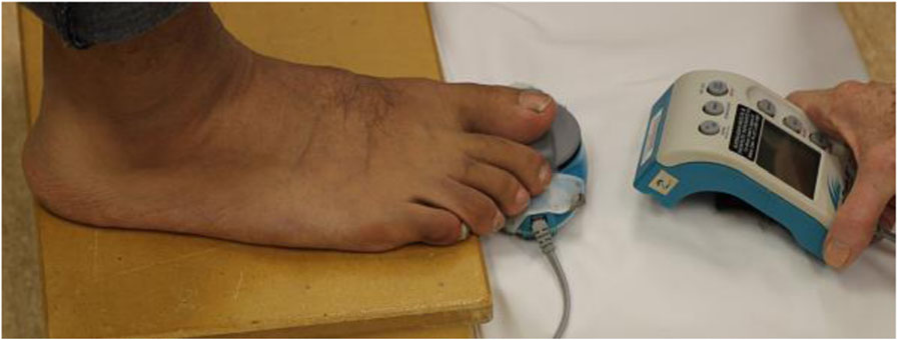
Dynamometry during the standing great toe press

Great toe flexion strength was assessed using a hand held dynamometer (Commander Muscle Tester, JTech Medical, Salt Lake City, UT USA). A customised support system was placed beneath the feet to maintain the foot and toes in a neutral position (Fig. 3). Testing was completed as per the procedure for the toe flexor task using the pressure platform. In standing, a secure bar was provided for participants to hold lightly to maintain balance while performing the task. Participants then kept the lower limb still while pressing as strongly as possible onto the force sensor of the hand held dynamometer [35].

#### Foot morphology

Foot alignment was measured using the Foot Posture Index (FPI), foot length (total and truncated) and dorsal arch height. The FPI consists of six criteria, [41] summed to provide a score from − 12 to + 12 for a supinated or pronated foot respectively with reported acceptable reliability [42].

Foot length and truncated foot length of the dominant foot was measured with the participant sitting in a chair with ankle, knees and hips flexed at 90°. Their feet were placed on a platform with an embedded ruler to measure full foot length from mid-heel to longest toe tip and truncated foot length from mid-heel to mid-first metatarsophalangeal (MTP) joint. Dorsal arch height in sitting and standing was measured with a digital height gauge with carbide scribe (Allendale Electronics Ltd., Hoddesdon Herts. UK). The gauge was placed at 50% of foot length to determine the Dorsal Arch Height (DAH) [43]. Arch Height Ratio (AHR) was determined by dividing the DAH by truncated foot length. This method has been shown to be a reliable and valid measure of arch height [44]. Foot arch mobility was determined by subtracting standing weight bearing dorsal arch height from sitting dorsal arch height [43].

#### Balance

Functional balance was tested with the maximal step length test. This test is a reliable predictor of mobility, balance and fall risk [45]. Participants stood behind a cross taped on the floor, with arms folded across the chest and palms up. They stepped with each leg (right then left) and in each direction (forward, side, back) as far as possible; paused while distance was recorded, then returned to the starting position. The standing foot remained firmly planted [46]. Distance was recorded only if balance and body posture were maintained throughout the test. Balance of the dominant leg was determined by averaging the total length stepped in each direction.

### Statistical analysis

Analysis was performed in SPSS for Windows v22.0 (IBM SPSS Inc., Chicago, IL). Intra-rater reliability of the variables was assessed with intraclass correlation coefficients (ICC_3,1_). Kappa was used to evaluate the Paper Grip test, with values ≤0 indicating no agreement and 0.01–0.20 none to slight, 0.21–0.40 fair, 0.41–0.60 moderate, 0.61–0.80 substantial, and 0.81–1.00 as almost perfect agreement [47]. Correlation analyses between intrinsic foot muscle size and anthropometrics (age, weight, height, BMI) foot morphology (foot length, truncated foot length, FPI, arch height), strength measures (hallux force by pedobarography and dynamometry) and balance (maximal step length test) were conducted with Pearson’s correlation coefficient. To account for the influence of physical body size a partial correlation was performed. The controlling variable was selected based on the variable with the highest and most consistent Pearson’s correlation coefficient for both AbH and FHBM muscles.

## Results

Participants were aged 39.5 ± 10.0 years (range 26–64 yrs.); female (15/21), BMI (23.8 ± 3.3 range 19-30Kg/m^2^), right foot dominant (19/21), FPI + 2.6 ± 1.5, (FPI of 2.4 ± 2.3 for adults is considered normal [48]), with Arch height flexibility .35 mm (Table 1). Due to low body weight, one participant’s data was excluded from all pedobarographic analysis as they were unable to generate acceptable force.

**Table 1:** Participant characteristics of the sample (*n* = 21)

Variable	^a^Value
*Participant characteristics*	
Age (y)	39.5 ± 10.0
Sex, Female (%)	15 (71%)
Body weight (kg)	65.5 ± 12.6
Height (m)	1.65 ± 0.08
BMI (kg/m^2^)	23.8 ± 3.3
Dominant foot, right	19 (90%)
*Foot morphology*	
Foot Posture Index (− 12 to 12 score)	2.6 ± 1.5
Foot length (cm)	24.2 ± 1.3
Truncated foot length (cm)	17.5 ± 0.91
Arch Height – sit (cm)	6.97 ± .75
Arch Height – stand (cm)	6.62 ± .74
Arch Height – mobility (cm)	.35 ± .17

^a^Values: mean ± SD

*Key: y* year, *kg* kilogram, *m* metres, *BMI* body mass index*, cm* centimetres

Intra-rater reliability for the ultrasound measures of cross-sectional area were excellent for AbH and FHBM (Table 2). The standing paper grip test had a Kappa value of 0.203, (*p* = 0.148) which is considered only slight reliability [49].

**Table 2:** Reproducibility of ultrasound cross-sectional area, pedobarography, hand-held dynamometry and balance measures

Variable	Trial 1 (mean ± SD)	Trial 2 (mean ± SD)	ICC_3,1_	95% CI	
*Ultrasound (cm* ^*2*^ *)*					
CSA Abductor Hallucis	2.16 ± 0.60	2.16 ± 0.63	0.97	0.94	0.99
CSA Flexor Hallucis Brevis	1.45 ± 0.35	1.45 ± 0.36	0.96	0.90	0.98
*Pedobarography (N)*					
Great toe press task (*n* = 20)					
Stand maximum force great toe	117.8 ± 33.8	128.1 ± 42.9	0.75	0.48	0.89
*Hand-held dynamometry (N)*					
Stand – great toe	124.9 ± 28.8	119.4 ± 28.3	0.75	0.48	0.89
*Balance (cm)*					
Mean maximal step right	89.3 ± 12.3	88.7 ± 12.37	0.83	0.63	0.93

*Key: ICC* Intraclass correlations coefficients, *CSA* Cross-sectional area, *cm* centimetres, *N* newtons

Notes-Pedobarography Emed Pressure Platform *n* = 20

Correlations between cross-sectional data are presented in Table 3. Positive significant associations were found between AbH cross-sectional area and the majority of participant characteristics (*r* = .502 to *r* = .625), arch height sitting (*r* = .597, *p* = .004), standing (*r* = .590, *p* = .005), toe flexion force using pedobarography (*r* = 623, *p* = .003) and maximum dominant step (*r* = .443, *p* = .044); and between FHBM cross-sectional area and foot length (*r* = .451, *p* = 040), truncated foot length (*r* = .483, *p* = .027) and FPI (*r* = .544, *p* = .011).

**Table 3:** Pearson’s correlations between ultrasound cross-sectional area and participant characteristics, foot morphology, pedobarography, hand-held dynamometry and balance measures

	Abductor Hallucis		Flexor Hallucis Brevis (Medial)	
Variable	*R*	*p*	*r*	*p*
*Participant characteristics*				
Age	0.070	0.763	−0.205	0.373
Weight	0.662**	0.001	0.305	0.179
Height	0.559*	0.008	0.372	0.097
BMI	0.502*	0.020	0.158	0.495
*Foot morphology*				
Foot length	0.582*	0.006	0.451*	0.040
Truncated foot length	0.580*	0.006	0.483*	0.027
Foot Posture Index	0.214	0.352	0.544*	0.011
Arch height sit	0.597**	0.004	0.062	0.790
Arch height stand	0.590**	0.005	0.089	0.702
*Hand-held dynamometry*				
Standing great toe force	0.011	0.964	−0.075	0.747
*Pedobarography*				
Stand max force great toe^a^	0.645**	0.002	0.349	0.132
*Balance*				
Maximum step Right	0.443*	0.044	0.356	0.113

*Key: BMI* Body mass index

^a^Missing data *n* = 20 **significant p < 0.005, * significant p < 0.05

Partial correlations controlled by truncated foot length are presented in Table 4. Positive significant partial correlations, were found between AbH cross-sectional area and toe flexion force using Pedobarography (*r* = 0.562, *p* = .012) and between FHBM cross-sectional area and the FPI (*r* = .631, *p* = .003).

**Table 4:** Partial Pearson’s correlations (controlling for truncated foot length) between ultrasound cross-sectional area and foot morphology, pedobarography, hand-held dynamometry and balance measures

	Abductor Hallucis		Flexor Hallucis brevis (medial)	
Variable	*r*	*p*	*r*	*p*
*Foot morphology*				
Foot Posture Index	0.275	0.240	0.631*	0.003
Arch height sit	0.403	0.078	−0.257	0.274
Arch height stand	0.437	0.054	−0.185	0.436
*Hand-held Dynamometry*				
Stand great toe force^a^	0.010	0.965	−0.087	0.714
*Pedobarography*				
Stand max force great toe^a^	0.562*	0.012	0.21	0.389
*Balance*				
Maximum step Right	−0.029	0.903	−0.046	0.848

*Abbreviations:* ^a^ Missing data n = 20 * significant p < 0.05

## Discussion

We found excellent reproducibility for ultrasound cross sectional area measures of AbH and FHBM while seated. Positive significant associations were found between the cross-sectional area of AbH and the majority of participant characteristics, toe strength determined by pedobarography, foot morphology; foot length and arch height, and balance. When controlling for truncated foot length, the association with toe strength determined by pedobarography remained consistent. Associations between the cross-sectional area of FHBM were limited to one foot morphology measure.

In this study the ultrasound transducer placement and position of participant was modified from previous studies on the reliability of ultrasound cross-sectional area measures [23, 25]. To maintain consistency of the seated ankle neutral position we scanned AbH by aligning with the navicular tubercle, this also ensured all three segments of the AbH muscle were imaged (Fig. 1a) [50]. As well as the impracticality of scanning the plantar aspect of the foot with the participant seated, variations in FHB anatomy influenced our scanning position. The lateral head of FHB is often inseparable from the oblique head of the adductor hallucis at the insertion [51] with difficulties in identifying the borders of FHB reported [52]. Furthermore, an anatomical cadaveric study has shown that 20% of insertions of the oblique head of adductor hallucis attach to the navicular and align with FHB lateral fibres [53]. Therefore, only the medial part of the FHB(M) muscle was scanned in the coronal plane on the medial-plantar aspect of the foot at about mid metatarsal in this study (Fig. 1b). This may explain the smaller cross-sectional area of FHBM from previously reported cross-sectional area FHB measures (Table 5) [23, 25, 54]. The participant was placed in seated ankle neutral for scanning both muscles to minimise any potential positional muscle size changes [30, 55]. The intra-rater reliability of the seated position and the scanning method of the AbH and FHBM was equivalent to previous studies [23, 25]. The excellent reliability of this approach suggests that for people with difficulty lying supine or prone, the seated position is a good alternative to determine cross-sectional area of these foot muscles.

**Table 5:** Literature review of cross-sectional area values for AbH and FHB (M) by ultrasound and MRI.

Author	Equipment	CSA AbH	Transducer alignment/region	CSA FHB	population	Transducer alignment/region
		Mean ± sd (cm²)		Mean ± sd (cm ²)		
Abe[59]	US	2.46±0.77	Medial hindfoot, inferior to medial malleolus	N/A	Sports active adults	
Angin[54]	US	2.75±0.34	Medial hindfoot, inferior to medial malleolus	2.97±0.46	Normal foot	Plantar, proximal forefoot thickest portion
		2.36±0.47		2.66 ±0.46	Pronated foot+8	
Battaglia[76]	US	2.47±0.93	Thickest portion from medial calcaneus distally towards the 1^st^ metatarsal	N/A	Healthy adults non w/b	
		2.60±0.91			Weight/bearing	
Lobo[61]	US	2.74± 0.64	Medial hindfoot thickest potion between medial calcaneal tuberosity and navicular tuberosity	2.13±0.65	Healthy adults no HV	Plantar mid forefoot thickest portion
		2.22± 0.49		1.57±0.41	Healthy adults with HV	
Mickle[20]	US	2.56±0.89	Medial hindfoot thickest portion between medial calcaneal tuberosity and navicular tuberosity	2.45±0.53	Healthy adults	Plantar, proximal forefoot thickest portion
		2.45±0.94	Medial hind foot inferior to medial malleolus			
Zhang[52]	US	2.62±0.56	Medial hindfoot, inferior to malleolus, thickest portion	Unable to determine	Runners; Normal foot	
		2.74±0.39			Pronated foot+ 6.6	
Current study	US	2.16±0.60	Medial, mid foot inferior to navicular tubercle thickest portion	1.44±0.35(M)	Healthy adults	Medial-plantar mid metatarsal thickest portion
Kura[72]	Muscle volume^*^	6.68±2.07		1.80± 0.75 FHB(M) 2.12± 0.84 FHBL		
		Total CSA: FHB and AbH				
Green[78]	MRI		3.00 mean			Medial foot
		Total CSA : FHB, FDB, Quadratus plantae, lumbricals and AbH				
Kurihara[31]	MRI		5.87±1.34			Forefoot 20% of Truncated foot length

^*^PCSA: Dissection, calipers and water displacement

Key: CSA: cross-sectional area, FHB: flexor hallucis brevis, AbH: abductor hallucis, M: medial, FDB: flexor digitorum brevis, AbH abductor hallucis, PCSA: physiological cross-sectional area, w/b: weight bearing, (M): medial

Cross-sectional area of AbH had significant associations with the majority of participant characteristics and foot morphology. Increasing body size was related to increasing AbH size. Associations between increased arch height and increased cross-sectional area of AbH was due to anatomical dimensions as the association became non-significant when controlling for truncated foot length. Also, the majority of participants had decreased arch flexibility according to McPoil and colleagues’ dorsal arch height norms [43]. However since arch height lowers with increased load [56] and with plantar muscle fatigue, [13, 57] the limited findings of the current study indicate maintenance of the height of the medial longitudinal arch may be more related to the cross-sectional area of AbH situated mid to hindfoot rather than the fore foot FHBM muscle.

In contrast, the cross-sectional area of FHBM had a substantially different pattern of association with strength, morphology and balance variables. A larger cross-sectional area of FHBM was significantly associated with a higher FPI (more pronated) even when controlled for truncated foot length. Zhang and colleagues reported a significantly larger AbH (> 4.3%) and flexor digitorum brevis (> 18.7%) associated with a more pronated FPI (6.6), [52] (Table 5) but they did not analyse FHB due to difficulty in identifying the muscle border. They proposed that the larger forefoot muscles of people with more pronated feet contribute to control of the forefoot abduction motion during gait. Interestingly, this contrasts with Angin and colleagues study comparing normal (FPI 1.3 ± 1.2) and pronated (FPI 8.1 ± 1.7) feet [54]. They report significantly smaller FHB (− 8.9%) and AbH (− 12%) in pronated feet compared to normal feet [54]. These varying findings regarding associations between AbH, FHB and flexor digitorum brevis cross-sectional area and their relationships with foot type, [52, 54] are similarly noted in studies examining intrinsic foot muscle size with age and gender, [58, 59] foot deformity [33, 60, 61] and plantar fasciitis [62, 63].

Some of the results of our study contrast with previous literature reporting positive associations between measures of cross-sectional area and toe flexion force [33, 58, 59, 64]. No association was found between cross-sectional area of either AbH or FHBM and toe flexor force measured by hand held dynamometry, which was unexpected. Previously, cross-sectional areas of intrinsic foot muscles determined by MRI were significantly correlated to measures of toe flexor strength using a toe grip dynamometer [31, 65]. Studies reporting good reliability for toe flexion used supported dynamometers with ICCs _3,1_ ranging from 0.931 [31] to 0.97 [2] or had participants braced or self-stabilised with ICC’s_3,1_ ranging 0.81 for hallux plantar flexion [66] to 0.95 for foot inversion [40]. The contrasting finding in our study may be due to the technique used to complete the hand held dynamometry measures in this study [67] (Fig. 3).

A significant association was found between cross-sectional area of AbH and great toe flexion strength measured by pedobarography. The positive relationship between increasing force and cross-sectional area was maintained even when controlling for physical dimensions, supporting previous findings [31, 65, 68]. This suggests that the cross-sectional area of AbH may be a useful early biomarker for foot muscle weakness. In contrast, no association was found between cross-sectional area of FHBM and toe flexion force. Muscle architecture, including shape and pennation angles, reaction time, innervation, fibre type and size, influences muscle force [69–72]. Ledoux [71] reported more than double pennation in AbH, which Tosovic and colleagues suggest has three segments, with each segment acting differently due to their pennate angle and fibre type [50, 71]. Furthermore, conflicting reports of forefoot or hindfoot muscle weakness in runners with plantar fasciitis [3, 62, 63] and the complexity of intrinsic foot muscle weakness associated with claw toes [60] suggests we may need to consider differentiation between fore, mid and hindfoot muscles when examining toe flexion strength related to foot problems.

Variations in muscle cross-sectional area or toe flexion force could be due to gender differences [73] or age related sarcopenia [50, 74]. Research to acquire the reference values for ultrasound cross-sectional area of various lower limb and foot muscles reported significant effects of age and sex on muscle thickness and echogenicity, [32] associated with fat infiltration [75]. We found a significant association between the size of AbH and sex, with males generally having a larger AbH, but no association between age and AbH or FHBM muscle size. Mickle and colleagues [58] reported significant age related difference between selected intrinsic and extrinsic foot muscles. They found significant differences in toe flexion force and FHB cross-sectional area but no significant difference in AbH or flexor digitorum brevis cross-sectional area between young and older participants. Change or reduction in muscle size may also be due to stance, [76] or loss of muscle fibres as well as decline in muscle fibre size, specifically type-II muscle fibres [75, 77]. The difference in patterns of association between cross-sectional areas of the AbH and FHBM muscles, foot morphology and toe flexion force may be due to the small number of participants evaluated in this study, the scanning positions used, as well as the architecture of the foot.

Balance, tested via maximal step length [45] was found to be significantly associated with AbH of the dominant leg. This suggests a positive relationship between muscle size and balance, somewhat supporting previous research, [16] and our hypothesis that a greater cross-sectional area of AbH and FHBM would be associated with better balance. Since only the size of the AbH was positively associated with toe flexion force, it is likely that strength of the AbH muscle plays a more important role in maintaining balance than FHBM. This result is also consistent with reports of increased activity of the abductor hallucis, flexor digitorum brevis and quadratus plantae muscles during a more demanding balance task [12]. However the relationship between AbH size and balance was not maintained after controlling for physical body size. This finding, along with the foot morphology results, highlights some associations may be entirely dependent on anthropometric variations.

There were several limitations to this study. First, only 21 healthy adults were evaluated from a sample of primarily female middle-aged adults, with less mobile or stiffer arched feet (Table 1), reducing the generalisability of the findings. Further, the small sample size resulted in a lack of statistical power with the possibility of Type 1 errors occurring as multiple comparisons were performed. Second, as this was a cross-sectional study no causality can be inferred. Third, only two muscles were measured in this study limiting comparisons with studies evaluating other intrinsic foot muscles.

## Conclusion

Measuring the cross-sectional area of AbH and FHBM muscles with ultrasound in the seated position is reproducible. Measures of toe flexion strength determined by pedobarography, foot morphology and balance appear to relate more to cross-sectional area of AbH than FHBM. While the first ray muscles may act as a unit, these forefoot and hind foot muscles exhibit different patterns of association between the variables. After controlling for physical body size, cross-sectional area of AbH remains a significant correlate of great toe flexor strength.

## Abbreviations

AbH: Abductor hallucis muscle
CSA: Cross-sectional area
FDB: Flexor digitorum brevis
FHB: Flexor hallucis brevis muscle
FHBM: Flexor hallucis brevis medial muscle belly
HV: Hallux valgus
ICC: Intra-class correlations
